# The Contribution of Thalamic Nuclei in Salience Processing

**DOI:** 10.3389/fnbeh.2021.634618

**Published:** 2021-02-16

**Authors:** Kuikui Zhou, Lin Zhu, Guoqiang Hou, Xueyu Chen, Bo Chen, Chuanzhong Yang, Yingjie Zhu

**Affiliations:** ^1^Shenzhen Key Laboratory of Drug Addiction, CAS Key Laboratory of Brain Connectome and Manipulation, The Brain Cognition and Brain Disease Institute (BCBDI), Shenzhen Institutes of Advanced Technology, Chinese Academy of Sciences, Shenzhen-Hong Kong Institute of Brain Science-Shenzhen Fundamental Research Institutions, Shenzhen, China; ^2^Department of Neonatology, Shenzhen Maternity & Child Healthcare Hospital, The First School of Clinical Medicine, Southern Medical University, Shenzhen, China

**Keywords:** salience, motivated behaviors, thalamus, paraventricular thalamus, mediodorsal thalamus

## Abstract

The brain continuously receives diverse information about the external environment and changes in the homeostatic state. The attribution of salience determines which stimuli capture attention and, therefore, plays an essential role in regulating emotions and guiding behaviors. Although the thalamus is included in the salience network, the neural mechanism of how the thalamus contributes to salience processing remains elusive. In this mini-review, we will focus on recent advances in understanding the specific roles of distinct thalamic nuclei in salience processing. We will summarize the functional connections between thalamus nuclei and other key nodes in the salience network. We will highlight the convergence of neural circuits involved in reward and pain processing, arousal, and attention control in thalamic structures. We will discuss how thalamic activities represent salience information in associative learning and how thalamic neurons modulate adaptive behaviors. Lastly, we will review recent studies which investigate the contribution of thalamic dysfunction to aberrant salience processing in neuropsychiatric disorders, such as drug addiction, posttraumatic stress disorder (PTSD), and schizophrenia. Based on emerging evidence from both human and rodent research, we propose that the thalamus, different from previous studies that as an information relay, has a broader role in coordinating the cognitive process and regulating emotions.

## Introduction

Complex sensory inputs about the external world and constant update of the internal state are fed into our neural system at every moment. The ability to capture the most relevant information from the noisy background is critical to both learning and survival. The saliency of a stimulus is not only determined by its physical properties but also influenced by different behavioral context and motivational states (Puglisi-Allegra and Ventura, 2012). Therefore, salience processing requires the cooperation of sensory, emotion, and attention systems throughout the brain (Uddin, 2015; Peters et al., 2016).

A variety of neuroimaging studies have revealed the cortical nodes of the salience network, the dorsal anterior cingulate cortex (dACC), and anterior insula (AI) (Downar et al., 2000, 2001; Seeley et al., 2007). Subcortical structures including the thalamus, the striatum, and the midbrain dopamine nuclei, which cooperate with the cortical nodes in cognitive control, also contribute to salience processing (Menon, 2011; Yeo et al., 2011; Wolff and Vann, 2019). Thalamic nuclei have reciprocal connections with the cerebral cortex and subcortical structures, participating in the regulation of arousal, emotion, and cognitive attention control (Schmahmann, 2003; Wolff and Vann, 2019). In the current mini-review, we will discuss the potential mechanism how the thalamus contributes to salience processing. Functional connectivity profiles of thalamic nuclei could throw light on how the thalamus might coordinate cortical and subcortical activities. Recordings of thalamic activities during adaptive behaviors provide critical evidence on how thalamic neurons encode salience and hence contribute to associative learning. We will also review recent evidence showing that dysfunction of thalamic nuclei is implicated in neural pathologies.

## Connectivity Profiles of Thalamic Nuclei

Early ideas about thalamic anatomy and function were derived from classic studies of the LGN (lateral genicular nucleus) (Hubel and Wiesel, 1962). LGN neurons receive topographic input from the retina and exhibit highly topographic projections and specific laminar patterns of terminations in the primary visual cortex. Therefore, it was widely believed that the thalamus is responsible for precise “bottom-up” transmission of input to primary cortical targets. In fact, this traditional view merely represents the anatomy and function of a restricted group of thalamic nuclei; other thalamic nuclei show distinct projection patterns, which are also categorized as non-specific thalamic nuclei (Kamikawa et al., 1967; Van der Werf et al., 2002; Vertes et al., 2015). For example, axon collaterals of neurons in the paraventricular thalamus (PVT) extend to the prefrontal cortex (PFC), nucleus accumbens (NAc), bed nucleus of the stria terminalis (BNST), and central amygdala (CeA), allowing simultaneous activation of distant brain regions (Bubser and Deutch, 1998; Dong et al., 2017; Millan et al., 2017). Non-specific thalamic nuclei, such as the midline and intralaminar thalamus, which do not receive direct input from ascending tracts but have diffuse projections to limbic cortical areas, hypothalamus and striatum (Kuramoto et al., 2015, 2017), will be the main focus of the current review.

Thalamocortical projections exhibit interesting anatomical features, which support the role of the thalamus in orchestrating cortical activities and regulating cognitive functions. For example, single neurons in the mediodorsal thalamus (MD) send axons to multiple prefrontal areas and form patchy axon arbors. This organization allows MD neurons to recruit a specific set of cortical neurons in distant cortical regions, compatible with the role of the MD in coordinating task-relevant cortical representations. In addition, thalamic axon arborizations are found not only in the relay layer (layer4) but also in the superficial layers of the cerebrocortex (Avendano et al., 1990; Rubio-Garrido et al., 2009), which are essential for information transmission and cortical computation. Another key feature of the thalamocortical pathway is the robust feedforward inhibition mediated by cortical inhibitory neurons (Cruikshank et al., 2007, 2010; Bagnall et al., 2011). A recent study reported that two thalamic nuclei target distinct types of cortical interneurons in the PFC and thus have differential influence on dendritic and somatic activity (Anastasiades et al., 2020). These properties are well equipped for temporal precise and pathway-specific regulation of cortical output. Besides reciprocal connections with the cerebral cortex, the thalamus receives extensive input from the brainstem and hypothalamus, obtaining information about general arousal and interoceptive states (Sherman, 2007; Kumar et al., 2017). The thalamus also projects to the striatum and amygdala, supporting its role in orientating motivation and regulating emotion.

Altogether, the thalamus is in position to detect and orientate neural resources toward behavioral relevant stimuli. This view is further evidenced by myriad functional imaging studies (Robinson and Petersen, 1992; Peters et al., 2016). On top of that, emerging circuitry studies investigating thalamic control of awareness and cognitive process have brought to light the mechanism how the thalamus contributes to salience control (Floresco and Grace, 2003; Halassa et al., 2014; Rikhye et al., 2018). In the subsequent sections, we will highlight recent advances in understanding the role of thalamic pathways in arousal and attention control, pain and reward, and emotion regulation.

### Thalamic Pathways Regulating Arousal State

Salience processing is often associated with amplification of certain sensory inputs and enhanced functional connectivity, whereas arousal also requires enhanced brain excitability and connectivity, but at a more general scale (Massimini et al., 2005; Nakajima and Halassa, 2017). Therefore, salience attribution could be conceptualized as dynamic control of specific arousal states of task-related neural circuits (Sakai, 2008). We will review evidence that thalamic circuitry participates in arousal regulation, aiming to give some clues on how the thalamus might contribute to salience processing.

Midline thalamic nuclei receiving hypothalamic and brainstem inputs connect with widespread cortical areas (Herkenham, 1979; Kuramoto et al., 2015). Hence, midline thalamic nuclei are well positioned anatomically to summate subcortical arousal information and modulate forebrain activity strongly and diffusely (Matyas et al., 2018; Ren et al., 2018). The PVT receives input from the brainstem arousal nuclei, such as the locus coeruleus and reticular formation (Krout et al., 2002; Li and Kirouac, 2012). Moreover, it is reciprocally connected with the suprachiasmatic nucleus (Alamilla et al., 2015; Yuan et al., 2018), which is the primary circadian pacemaker in the brain. Moreover, the PVT is also densely innervated by orexinergic fibers (Matzeu et al., 2014), the activation of which depolarizes postsynaptic PVT neurons (Ishibashi et al., 2005). Compelling evidence has demonstrated a key role of the orexin/hypocretin system in arousal and maintenance of the awaking state (de Lecea, 2012). Activation of the PVT effectively promotes wakefulness under the regulation of hypocretin neurons in the lateral hypothalamus (Ren et al., 2018). Moreover, different PVT output pathways might be in charge of arousal regulation in various homeostatic states (Hua et al., 2018; Meffre et al., 2019). However, a recent study found a subpopulation of PVT neurons that are negatively modulated by wakefulness and arousal (Gao et al., 2020). This discrepancy might rise from the difference in anatomical location that previous studies focused on the posterior part of the PVT, whereas the recent one investigated a genetically defined subgroup of neurons which populates in the anterior part. Future studies examining the afferents of distinct PVT subregions and subpopulations will help clarify the role of the PVT in arousal.

The majority of thalamic neurons are glutamatergic; however, neurons in the reticular thalamus (TRN) are primarily GABAergic and exert inhibitory control over thalamic nuclei (Halassa and Acsady, 2016). Spontaneous firing of midline thalamic neurons in mice is phase-advanced to global cortical up states (Gent et al., 2018). Whereas enhanced spiking during sleep is found in TRN subnetworks that project to sensory-related thalamic circuits (Halassa et al., 2014; Chen et al., 2015b), attention decreases TRN responses to visual stimuli (McAlonan et al., 2008). The TRN regulates thalamocortical activities and has a causal role in generating sleep spindles (Bazhenov et al., 2000; Cueni et al., 2008; Halassa et al., 2011). Optogenetic manipulations of TRN activities bidirectionally modulate arousal states (Lewis et al., 2015; Herrera et al., 2016). Recent studies have revealed that cholinergic and noradrenergic inputs to the TRN participate in regulating sleep and arousal (Ni et al., 2016; Zhang et al., 2019).

### Pain- and Reward-Related Thalamic Pathways

Neurocircuits regulating pain and reward have been considered as part of the system orientating attention toward various salient stimuli (Roland, 1992; Uddin, 2015; Kummer et al., 2020). Thalamic neurons receiving inputs from the spinal cord, the midbrain, and the hypothalamus (Yen and Lu, 2013; Kirouac, 2015) are activated by noxious and appetitive stimuli (Casey and Morrow, 1983; Kim et al., 2003). The medial and intralaminar thalamic nuclei are the major source of pain-associated information to the limbic cortex (Livneh et al., 2017; Meda et al., 2019; Liang et al., 2020a).

Inactivation of the dorsal thalamic nuclei has been shown to suppress pain and pain related aversion (Jurik et al., 2015; Cheng et al., 2017; Zhou et al., 2019). In addition, the ratio between excitation and feedforward inhibition of thalamic input to the cortex is important for the regulation of affective pain (Jurik et al., 2015; Meda et al., 2019). Activation of the PVT-CeA pathway induces mechanical allodynia (Liang et al., 2020b), reflecting a potential role of the PVT in pain-associated salience attribution. The PVT is also engaged in reward-seeking behaviors (James et al., 2011a; Browning et al., 2014; Choudhary et al., 2018). The PVT shows increased neuronal activation in response to reward and reward-associated cues (Igelstrom et al., 2010; James et al., 2011a; Yeoh et al., 2014; Munkhzaya et al., 2020). Suppression of PVT activities could attenuate reward-motivated behaviors (Hamlin et al., 2009; Ong et al., 2017) (but see Stratford and Wirtshafter, 2013; Zhang and van den Pol, 2017). A growing body of evidence suggests that the PVT plays a role in integrating complex homeostatic signals and informing adaptive behaviors during motivational conflicts (Ferrario et al., 2016; Choi and McNally, 2017; Meffre et al., 2019). Using *in vivo* single-unit recording, Zhu et al. showed that posterior PVT neurons could be activated by both rewarding and aversive stimulus and the cues predicting those outcomes, indicating that the pPVT encodes stimulus salience irrespective of valance (Zhu et al., 2018). By alterations of the behavioral context and modulation of homeostatic states, they further demonstrated that the PVT provides dynamic representation of salience and thus contribute to associative learning.

### Thalamic Circuitry in the Regulation of Attention

By definition, salience describes the ability of a stimulus or an event to capture attention. On the other hand, salience attribution is strongly influenced by top-down attention control and emotional states. Thalamic nuclei, such as the anterior thalamic nuclei (ATN) and the MD, contribute to attention control primarily via their connections with limbic structures (Parnaudeau et al., 2013; Wright et al., 2015; Wolff and Vann, 2019). The ATN is densely connected with both the hippocampus and the frontal lobe and thus is believed to play a role in memory-guided attention (Leszczynski and Staudigl, 2016). Using deep brain stimulation (DBS) in human subjects, studies suggest that the ATN is involved in emotion–attention interaction (Hartikainen et al., 2014; Sun et al., 2015).

Accumulating evidence from human and animal studies have indicated that disruption of the MD impairs cognitive processes (Block et al., 2007; Nakajima and Halassa, 2017; Parnaudeau et al., 2018; Pergola et al., 2018). Anatomically, the MD receives modulatory input from the midbrain and brainstem and forms a reciprocal connection with the frontal cortex (Russchen et al., 1987; Mitchell, 2015; Collins et al., 2018). Single MD neurons receive convergence of small cortical inputs and project to multiple cortices across multiple layers (Rubio-Garrido et al., 2009; Kuramoto et al., 2017; Georgescu et al., 2020). Schmitt et al. (2017) proposed that the MD sustains rule representations in the PFC. Interestingly, enhancing MD excitability improved rule specificity and behavioral performance, in contrast to the reduction of rule information induced by enhancing PFC excitability. These results imply that the MD input exerts the effect by regulating the functional micro-circuitry in the PFC instead of non-selectively boosting the excitability of pyramidal neurons.

Dynamic control of salience is also reflected in the process of cognitive switching, which is important for action selection and behavioral flexibility. Thalamic nuclei are involved in cognitive switching primarily through connections with the striatum and PFC (Phillips et al., 2016). The centromedian (CM) and parafascicular nucleus (PF) are the major thalamic input to the dorsal striatum (DS) (Ilyas et al., 2019). Primate studies have suggested that the CM and PF provide the striatum with information about salience (Matsumoto et al., 2001; Minamimoto and Kimura, 2002; Yamanaka et al., 2018). Studies in rodents extend these findings by showing that disruption of CM/PF-DS pathway increased the perseverative responses and aggravated the interference between new and old learning (Bradfield et al., 2013; Bradfield and Balleine, 2017; Saund et al., 2017; Kato et al., 2018). The MD also contributes to behavioral flexibility and probably exerts its role through feedforward inhibition (Kuroda et al., 2004; Rotaru et al., 2005; Block et al., 2007; Delevich et al., 2015). In addition, a recent study suggested that MD-mediated suppression preserves unused cortical traces for future use (Rikhye et al., 2018). Subnetworks in the TRN exert inhibitory control over spatially discrete thalamic targets, suppressing distracting inputs (Pinault and Deschenes, 1998; Zikopoulos and Barbas, 2012; Halassa et al., 2014; Wimmer et al., 2015). Although the PFC does not directly project to the TRN, the PFC could regulate modality specific TRN subnetworks via the globus pallidus (GP) (Nakajima et al., 2019). In addition, the TRN is innervated by amygdalar input, providing a mechanism for emotion-driven attention shift (Zikopoulos and Barbas, 2012).

### Thalamic Circuitry in the Regulation of Emotion

Without adequate assignment of salience, stimuli that typically trigger mood and emotions can no longer attract one’s interest to act and react. Thus, it is not surprising that aberrant functional connectivity in the salience network is frequently observed in depressed patients (Pannekoek et al., 2014; Yuen et al., 2014; Rzepa and McCabe, 2016). Functional MRI studies have reported decreased functional connectivity between the dACC and the MD in the depressed patients (Wang et al., 2012). Moreover, improved MD-PFC connectivity has been associated with effective depression treatments (Salomons et al., 2014; Leaver et al., 2016). Consistently, synaptic strength of the MD-PFC pathway is reduced in a rodent model of depression, while activation of this pathway is sufficient to reduce depression-like behavior (Miller et al., 2017). Interestingly, a recent study found that the visual thalamus could affect the midbrain monoaminergic centers via the lateral habenula (Huang et al., 2019). In addition, this pathway might mediate the antidepressive effect of light therapy.

Thalamic nuclei modulate both innate fear and conditioned fear responses (Li et al., 2004; Penzo et al., 2015; Salay et al., 2018). Fear extinction is a process involving progressive suppression of the salience of fear-associative cue or context. Manipulations of thalamic activities have been shown to bidirectionally modulate fear extinction (Padilla-Coreano et al., 2012; Matyas et al., 2014; Paydar et al., 2014; Do-Monte et al., 2015; Lee et al., 2019; Ramanathan and Maren, 2019). Thalamic neurons exhibit two firing modes, tonic and burst, which could modulate behaviors in opposite directions (Sherman, 2001). Using tetrode recording in free-moving mice, Lee et al. (2011) showed that the tonic firing frequency of MD neurons positively correlates with the extent of fear extinction. In addition, enhancing tonic firing of MD neurons facilitated fear extinction, whereas burst-evoking stimulation suppressed extinction, indicating that distinct firing modes of MD neurons might bidirectionally modulate salience of fear-associated cue (Lee et al., 2011; Georgescu et al., 2020).

On the other hand, augmented observational fear responses have been demonstrated in socially related conspecifics (Jeon et al., 2010). Social transmission of fear is associated with a significant increase of activity in the PVT, MD, and ACC (Chang and Debiec, 2016; Zheng et al., 2020). Since the PVT and MD have been reported to regulate social related behaviors (Zhou et al., 2017; Watarai et al., 2020; Yamamuro et al., 2020), they might participate in social salience modulation of fear response.

## Thalamic Dysfunction and Neuropsychiatric Disorders

Given the essential role of the thalamus in salience processing, it is perhaps not surprising that altered connectivity patterns and responses of thalamic nuclei have been poised to contribute to the aberrant salience attribution in neuropsychiatric disorders. Below, we will describe the engagement of thalamic dysfunction in three distinct mental disorders, in which dysregulation of salience processing is often observed.

### Drug Addiction Disorder

Drugs of abuse profoundly modulate neural response toward previous neutral stimuli which become associated with drugs. The development of incentive salience of drug-paired context or cues is an essential component of drug addiction (Koob and Volkow, 2016). As discussed above, the PVT is anatomically well positioned to coordinate drug-related behaviors (Browning et al., 2014; Zhou and Zhu, 2019). PVT neurons express orexin, opioid, and dopaminergic receptors and receive multiple neuromodulatory inputs (Mansour et al., 1986; Clark et al., 2017). PVT neuronal activity and plasticity in PVT-related pathways are modulated by drug-related behaviors (Deutch et al., 1998; Kolaj et al., 2014; Yeoh et al., 2014; Chen et al., 2015a; Zhu et al., 2016). Consistent with the idea that PVT neurons encode stimulus salience, a recent study showed that manipulations of the PVT-CeA pathway could bidirectionally modulate morphine-conditioned place preference, suggesting that the PVT-CeA pathway associates incentive salience of the drug with paired environment (Keyes et al., 2020). However, manipulations of the PVT pathways could result in diverse outcomes in literature (Table 1). Further investigation of the heterogeneity in anatomical location, connectivity profile, activity pattern, and genetic markers of the PVT neurons might help to resolve this ambiguity (Millan et al., 2017; McGinty and Otis, 2020).

**TABLE 1:** Effect of PVT manipulations on drug related behaviors.

Position/pathway	Manipulation	Effect (↑ - ↓)		Drug	Species	References
aPVT	Baclofen and muscimol	**↓** Expression of CPP		Cocaine	Rat	Browning et al., 2014
aPVT	Orexins	**↑** Drug consumption		Ethanol	Rat	Barson et al., 2015
	Ox2R antagonist	**↓** Drug consumption				
aPVT	NTS	**-** Drug consumption in lower drinkers		Ethanol	Rat	Pandey et al., 2019
PVT	Electrolytic lesion	**↓** Locomotor activity and sensitization		Cocaine	Rat	Young and Deutch, 1998
PVT	Excitotoxic lesion	**-** Acquisition of drug seeking		Ethanol	Rat	Hamlin et al., 2009
		**↓** Context-induced reinstatement				
PVT	TTX or CART	**↓** Drug-primed reinstatement		Cocaine	Rat	James et al., 2010
PVT	Orxr1 antagonist	**-** Cue induced reinstatement		Cocaine	Rat	James et al., 2011b
PVT	Baclofen and muscimol	Cue-induced drug-seeking in goal-trackers**↓**; in sign-trackers **-**		Cocaine	Rat	Kuhn et al., 2018
pPVT	OX2R antagonist	**↓** Naloxone-precipitated CPA		Morphine	Rat	Li et al., 2011
	OX1R antagonist	**-** Naloxone-precipitated CPA				
pPVT	Orexins/OX1R or OX2R antagonist	**-** Drug consumption		Ethanol	Rat	Barson et al., 2015
pPVT	Orexin A	**↑** Cocaine reinstatement		Cocaine	Rat	Matzeu et al., 2016
	OX2R antagonist	↓ Orexin primed reinstatement				
	OX1R antagonist	**-** Orexin primed cocaine seeking				
pPVT	Overexpression of D2Rs	↓ Locomotor sensitization		Cocaine	Mouse	Clark et al., 2017
pPVT	Orexin A	**↑**	Drug	Cocaine	Rat	Matzeu et al., 2018
	Dynorphin	**-**	reinstatement			
	Orexin A and Dyanorphin	**↓** Orexin-induced drug reinstatement				
pPVT	NTS	**↓** Drug consumption in higher drinkers		Ethanol	Rat	Pandey et al., 2019
	NTS antagonist	**↑** Drug consumption				
PVT→ NAc	Tetanus toxin	↓ Self-administration		Cocaine	Rat	Neumann et al., 2016
		**-** Incubation of craving after prolonged withdrawal				
pPVT→ NAc	Chemogenetic or optogenetic inhibition/optogenentic LTD	**↓** Naloxone-precipitated withdrawal symptoms **↓** CPA		Morphine	Mouse	Zhu et al., 2016
PrL→ PVT	Chemogenetic inhibition	**↓** Context or cue induced drug reinstatement		Cocaine	Rat	Giannotti et al., 2018
pPVT→ NAc	Chemogenetic/optogenetic inhibition	**↓** Retrieval of CPP; **-** Acquisition of CPP		Morphine	Mouse	Keyes et al., 2020
pPVT→ CeA	Chemogenetic inhibition	**↓** Acquisition of CPP;				
		**-** Retrieval of CPP				
	Optogenentic activation	**↑** CPP at suboptimal dose				
pPVT	Baclofen and muscimol	**-**		Heroin	Rat	Chisholm et al., 2020a
	Chemogenetic activation	**↓**				
PrL→ pPVT	Chemogenetic activation or inhibition	**-**	Food restriction-induced augmentation of drug seeking			Chisholm et al., 2020b
pPVT→ NAc core	Chemogenetic activation	**-**				
pPVT→ NAc shell	Chemogenetic activation	**↓**				
						

The phenomenon that drug-seeking behaviors progressively increase after abstinence is termed incubation of craving (Lu et al., 2004; Pickens et al., 2011). In addition to the contribution of the mesolimbic dopaminergic signal (Caprioli et al., 2017; Rossi et al., 2020), glutamate has been shown to participate in incubation of craving (Li et al., 2015; Shin et al., 2016). Incubated methamphetamine (Meth) seeking selectively activated glutamatergic input from anterior intralaminar nuclei of the thalamus (AIT) to dorsomedial striatum (DMS) (Li et al., 2018). Furthermore, inactivation of the AIT-DMS pathway attenuated incubated Meth craving while leaving non-incubated Meth seeking intact. These results suggest a critical role of the AIT in the regulation of incentive salience and drug relapse.

### Posttraumatic Stress Disorder

Posttraumatic stress disorder (PTSD) is a long-lasting and recurring mental disorder triggered by traumatic experience. Trauma recollection is associated with enhanced connectivity in the salience network, while salience connectivity is reduced following effective treatment in PTSD patients (Abdallah et al., 2019a, b). PTSD subjects showed significantly less activation of the thalamus (Lanius et al., 2001; Suarez-Jimenez et al., 2020). In a case report, new onset of PTSD occurred after thalamic infarct in a Korean War veteran (Duggal, 2002). In addition, an fMRI study showed that a larger magnitude of spontaneous activity in the thalamus is associated with lower reexperiencing symptoms in PTSD (Yan et al., 2013). Studies in rodent animals further analyzed the requirement of thalamic function during different time points of fear retrieval. For example, the PVT is gradually recruited during fear retrieval (Padilla-Coreano et al., 2012). Suppression of the PVT-CeA pathway disrupts fear retrieval at late but not early time points, suggesting the induction of long-term plasticity in this pathway (Do-Monte et al., 2015; Penzo et al., 2015).

Psychotherapeutic strategies for treating PTSD often involve reassignment of salience and modulation of attentional processes (Badura-Brack et al., 2015). For example, eye movement desensitization and reprocessing (EMDR) is a treatment using alternating bilateral sensory stimulation (ABS) to interfere with fear memory recall (Novo Navarro et al., 2018). The mechanism underlying the therapeutic effect of visual ABS can be inferred from the study by Baek et al. They found that ABS could drive activity in the superior colliculus (SC)-MD pathway which induces sustained BLA inhibition during fear extinction (Baek et al., 2019). Their results argue that MD mediates the competition between visual–attentional process and emotional activity and serves as a key target of treatment for PTSD.

### Schizophrenia

Schizophrenia is a mental disorder involving a range of problems in cognition, emotion, and behaviors. One influential theory about schizophrenia is that positive symptoms including hallucination and illusion could be attributed to aberrant assignment of salience to a certain experience or internal representation (Kapur, 2003; van Os and Kapur, 2009; Kim et al., 2018).

Abnormalities in structure and function of the thalamus have been associated with schizophrenia (Byne et al., 2009). According to genome-wide association studies, thalamic neurons express several schizophrenia-relevant genes (Watis et al., 2008; Pergola et al., 2015; Takahashi et al., 2015; Krol et al., 2018). A reduced volume of the thalamus has been found in schizophrenia patients (Volz et al., 2000; Konick and Friedman, 2001). A decreased number of parvalbumin (PV) neurons in the thalamus have been reported in human postmortem schizophrenia brains and rodent models (Danos et al., 1998; Steullet et al., 2018). Studies indicate that thalamocortical connections are compromised in schizophrenia (Sharp et al., 2001; Woodward et al., 2012; Avram et al., 2018; Delevich et al., 2020), which might cause disturbances in sensory gating and top-down control (Anticevic et al., 2014).

## Future Directions

As discussed above, thalamic neurons could encode salient features of stimuli, contributing to cognitive process and emotional regulation. However, it remains unknown how the information of salience is integrated from distinct pathways in single thalamic neurons. Also, it is worth further investigating the role of the neuromodulatory system on salience control in the thalamus. Stress induces perturbations in the structure and function of the brain, which present a major risk factor for many neuropsychiatric disorders. Future studies describing the molecular and circuitry adaptations in acute and chronic stress, would help us to analyze the contribution of the thalamus to salience allocation under pathological conditions.

## Author Contributions

KZ, LZ, and GH contributed equally to the writing of this review article. All authors contributed to the article and approved the submitted version.

## Conflict of Interest

The authors declare that the research was conducted in the absence of any commercial or financial relationships that could be construed as a potential conflict of interest.
